# Component resolved diagnosis in baker's asthma

**DOI:** 10.1186/2045-7022-4-S2-P35

**Published:** 2014-03-17

**Authors:** Cristina Gómez-Casado, María Garrido-Arandia, Catia Pereira, Ana Aranda, Manuela Catarino, Alicia Armentia, Santiago Quirce, Paloma Campo, Araceli Díaz-Perales

**Affiliations:** 1 CBGP (UPM-INIA) Technical University Madrid, CBGP (UPM-INIA) Campus Montegancedo, Autovía M-40 Salida 38N 36S, Pozuelo de Alarcón (Madrid), 28223, Spain; 2 Technical University CBGP (UPM-INIA), Pozuelo de Alarcón (Madrid), Spain; 3 University of Lisbon, Faculty of Pharmacy, Lisbon, Portugal; 4 Civil Hospital of Malaga, IMABIS Foundation, Malaga, Spain; 5 Rio Ortega University Hospital, Allergy Service, Valladolid, Spain; 6 La Paz University Hospital, Allergy Service, Madrid, Spain

## Background

Baker's asthma is one of the most common types of occupational asthma and its prevalence is increasing in the last years. Diagnosis of occupational asthma is complex. The poor specificity of current diagnostic approaches may be associated with insufficient purity of wheat extracts or lack of inclusion of major allergens in them. In this work, we use microarray technology to characterize the allergenic profiles of baker's asthma patients from three regions in Spain and to analyze the influence of other environmental allergens on the sensitization pattern.

## Methods

A panel of wheat allergens was purified from natural sources and printed on a protein microarray by standard methods. Additionally, representative aeroallergens were included in the protein microarray. Individual sera from three groups of patients (baker's asthma-BA-, seasonal rhinitis-SR- and wheat food allergy-WFA-) and three regions in Spain (Madrid, Malaga and Valladolid) were incubated with the protein microarray and compared.

## Results

WTAI-CM16 and Tri a 14 were the most prevalent allergens in the BA group (54 and 45%, respectively), covering a total of 64% of the BA population. Patients with seasonal rhinitis (SR) were not sensitized to wheat allergens. Tri a 14, the wheat LTP, was only recognized by BA patients. Although geographically different environmental allergenic load existed in the three regions, BA sensitization profiles were comparable.

## Conclusion

Baker's asthma is a disease associated with the daily handling of wheat flour, regardless of local aeroallergens.

